# Engaging in an experiential processing mode increases positive emotional response during recall of pleasant autobiographical memories

**DOI:** 10.1016/j.brat.2017.02.005

**Published:** 2017-05

**Authors:** Darius Gadeikis, Nikita Bos, Susanne Schweizer, Fionnuala Murphy, Barnaby Dunn

**Affiliations:** a MRC Cognition and Brain Sciences Unit, Cambridge, UK; b Maastricht University, Maastricht, The Netherlands; c Mood Disorders Centre, University of Exeter, UK

**Keywords:** Processing mode, Positive affect, Autobiographical memory, Emotion regulation, Anhedonia, Happiness

## Abstract

It is important to identify effective emotion regulation strategies to increase positive emotion experience in the general population and in clinical conditions characterized by anhedonia. There are indications that engaging in experiential processing (direct awareness of sensory and bodily experience) bolsters positive emotion experience but this has not been extensively tested during memory recall. To further test this notion, 99 community participants recalled two positive autobiographical memories. Prior to the second recall, participants either underwent an experiential, analytical, or distraction induction (n = 33 per condition). Subjective happiness and sadness ratings and heart rate variability (HRV) response were measured during each recall. Greater spontaneous use of experiential processing during the first memory was associated with greater happiness experience, but was unrelated to HRV and sadness experience. Inducing experiential processing increased happiness experience relative to both the analytical and distraction conditions (but had no impact on sadness experience). There was a significant difference in HRV between conditions. The experiential condition led to a trend-significant increase, and the other conditions a non-significant decrease, in HRV from the first to the second memory. These results suggest that engaging in experiential processing is an effective way to up-regulate positive emotion experience during positive memory recall.

## Highlights

• Experiential processing mode was induced during positive memory recall.
• Experiential processing mode increased happiness but not sadness.
• Inducing experiential processing therefore up-regulates positive emotion experience.

## 1 Introduction

There is increasing interest in understanding positive emotion regulation, defined as the range of processes used to change the nature, frequency and intensity of positive emotion experience (Bryant et al., 2011, Carl et al., 2013, Quoidbach and Gross, 2015). Helping individuals enhance positive emotions in appropriate situations may increase wellbeing in the general population and in clinical groups who experience anhedonia (e.g., depression, social phobia and schizophrenia; Dunn, 2012, Dunn and Roberts, 2016, Kashdan et al., 2011, Watson and Naragon-Gainey, 2010).

The way in which individuals direct their attention during potentially positive activities arguably impacts their experience of positive emotions. In particular, several therapeutic approaches emphasize the value of attending to sensory and bodily experience to amplify positive affect. Behavioral activation approaches utilise ‘attention to experience’ exercises to encourage individuals to repeatedly direct attention to external sensory experience (Dimidjian et al., 2011, Martell et al., 2010). Imagery techniques encourage individual to generate vivid, rich representations of experience incorporating sensory information in the mind's eye (Holmes, Blackwell, Burnett Heyes, Renner & Raes, 2016), likening this to “weak perception” (Pearson, Naselaris, Holmes, & Kosslyn, 2015). Mindfulness interventions attempt to cultivate a ‘being’ mode, where individuals attend to sensory and bodily experience without judgement as it unfolds in the moment (Segal et al., 2002, Teasdale, 1999; Williams, 2008). Positive psychology savouring techniques include promoting experiential absorption, where an individual engrosses themselves in perceptual experience and focuses their attention on the most positive aspects of this experience (sensory-perceptual sharpening; Bryant et al., 2011, Bryant and Veroff, 2007). Collectively, these techniques can be described as fostering an experiential processing mode (characterized by direct, non-judgemental and concrete awareness of sensory and bodily experience as it unfolds moment-to-moment).

A growing body of evidence is now starting to converge on the finding that engaging in an experiential processing mode bolsters positive affective experience. For example, in the imagery domain a range of studies have shown that imagining things in the mind's eye as opposed to thinking about things verbally tends to enhance positive affective experience (Holmes, Lang, & Shah, 2009; Holmes, Mathews, Dalgleish, & Mackintosh, 2006). Moreover, two recent clinical trials demonstrate that imagery training can help repair anhedonia in the context of depression (Blackwell et al., 2015, Pictet et al., 2016). While the beneficial effects of experiential processing on positive mood have not always been replicated using alternative induction techniques (e.g. see Hetherington & Moulds, 2013), the consensus view is that experiential processing during engagement with a potentially positive activity is likely to bolster positive mood.

However, as far as we are aware, there is less consensus on the consequences of engaging in an experiential processing mode on positive emotions when *recalling* a positive experience. There is an increasing interest in the field of ‘memory therapeutics’ (Dalgleish & Werner-Seidler, 2014) and it is likely to be of clinical benefit to help individuals be able to recall, and re-experience, pleasant memories in a variety of psychological conditions.

A handful of studies have made use of well validated procedures to induce an experiential processing mode and assess its cognitive-affective consequences (for example, see Watkins and Teasdale, 2001, Watkins and Teasdale, 2004, Watkins et al., 2008). In the typical procedure, participants are asked to read a series of self-referential sentences and focus on the sensory experience these bring to mind (referred to in the literature either as an experiential or concrete processing mode induction). This is contrasted to another condition where participants are asked to read the same series of sentences but to focus on the causes, meanings and consequences of them, so as to induce an evaluative, conceptual and abstract analysis of experience (referred to in the literature either as an analytical or abstract processing mode induction). Using this approach, three studies have examined if inducing particular processing modes changes how well individuals can use positive memory recall to repair negative mood after undergoing a negative mood manipulation (Hetherington and Moulds, 2015, Werner-Seidler and Moulds, 2012, Werner-Seidler and Moulds, 2014). However, the focus within two of these studies was changes in negative mood during the memory recall phase and changes in positive mood were not measured (Werner-Seidler and Moulds, 2012, Werner-Seidler and Moulds, 2014). Hetherington and Moulds (2015) did report positive affect ratings, unexpectedly finding that there was a greater increase in happiness experience following the induction of both an experiential and an analytical processing mode (relative to a distraction condition). All of these studies focus on positive emotion experience during memory recall following a negative mood manipulation and it is unclear if a similar pattern of findings would emerge during positive memory recall in a more neutral mood state.

To the best of our knowledge, only one study has examined the impact of processing mode on positive emotion experience during memory recall (unconfounded by a prior negative mood manipulation). Individuals were asked to recall a positive autobiographical memory whilst following concrete/imagery instructions (akin to an experiential processing mode) or abstract/verbal instructions (akin to an analytic processing mode) (Nelis et al., 2015; Study One). Findings revealed a greater increase in positive affect in those allocated to the concrete/imagery versus abstract/verbal induction. However, in a follow-up study within the same manuscript this effect was not replicated, with no significant difference emerging between the concrete/imagery and abstract/verbal conditions in this second study (Nelis et al., 2015; Study Two).^1^ Moreover, in the absence of a neutral control condition it is difficult to interpret whether the significant condition effect in Study One of Nelis et al. (2015) reflected the concrete/imagery condition increasing, or the abstract/verbal condition decreasing, positive emotion experience.

There are two other design issues with all of the extant studies examining links between experiential processing mode and positive emotional response during memory recall, which further complicate interpretation of the findings. First, positive emotion experience was assessed solely using self-report measures, which are vulnerable to demand characteristics (Nichols & Maner, 2008). Recording objective physiological measures of positive affect, which are less influenced by experimental demands, would strengthen conviction in the findings. For example, greater heart rate variability (HRV) has been linked to increased activation of the positive affect system (Kok and Fredrickson, 2010, Kok et al., 2013). HRV in part reflects functioning of the vagus nerve, a core part of the parasympathetic nervous system that regulates how fast the heart beats when an organism experiences signals of interest or safety and is believed to promote social-affiliative behavior (Porges, 2007, Appelhans and Luecken, 2006, Thayer and Lane, 2000). Second, all studies relied on a purely between-subjects design (participants underwent a single positive mood recall, having first been randomized to one of the experimental processing mode inductions). Individual differences in emotional response to the positive mood manipulation itself may have over-ridden any processing mode induction effects, reducing the sensitivity of these designs. A mixed within-between participants design (where individuals undergo a positive mood manipulation before and after processing mode is induced) can better control for individual differences in positive emotional experience between participants and may therefore have greater sensitivity. In particular, an analysis of covariance approach can be used, whereby response to the mood manipulation *before* the processing mode induction can be entered as a covariate in the model predicting response to the mood manipulation *after* the processing mode induction. Moreover, being able to examine changes in emotional experience from pre- to post- induction within participants makes it possible to establish more clearly if each condition increases or decreases positive emotion experience. A final advantage of a mixed within- and between-subjects design is the ability to assess if spontaneous use of each processing mode is related to positive emotional experience during the positive memory recall prior to inducing processing mode.

Therefore, the aim of the current study was to clarify whether inducing an experiential processing mode does bolster positive emotion experience during positive memory recall when using a mixed within-between-subjects design. Participants recalled two positive autobiographical memories before and after being randomized to either an experiential processing mode induction, an analytical processing mode induction, or a distraction induction (intended as a neutral control condition). We measured subjective happiness experience and HRV change during memory recall to index positive emotion experience. Our first hypothesis was that greater spontaneous use of an experiential processing mode during the first memory recall would be associated with greater happiness experience and greater HRV. Our second hypothesis was that experimentally inducing an experiential processing mode would result in increased happiness experience and greater heart rate variability during the second positive memory recall, relative to both the analytical and distraction comparison conditions. We had no *a priori* predictions about the association between spontaneous use of analytical processing and positive emotional response during the first memory recall, nor the impact of inducing analytical processing from the first to the second memory recall on positive emotional response. We additionally assessed sadness experience during memory recall to assess the impact of processing mode on negative affect.

## 2 Method

### 2.1 Participants

Ninety-nine community volunteers (aged 18–65) were recruited into the study from the Medical Research Council Cognition and Brain Sciences Unit (MRC CBU) panel of community volunteers in Cambridge, UK. The panel holds details of individuals aged greater than sixteen in the Cambridge area who are willing to be contacted to take part in research experiments. Participants were excluded if they reported a history of drug abuse, neurological problems or current mental health problems diagnosed by a health care practitioner, based on their responses to a series of semi-structured questions asked by the experimenter at the start of the experiment. We asked participants if they had any current experience of mental health difficulties. If they answered yes, this was followed up by a series of probe questions to establish if this was a diagnosis by a health care practitioner. The Cambridge Psychological Research Ethics Committee approved the study and participants provided written informed consent (initially via e-mail correspondence prior to the testing session and confirmed in person during the testing session).

### 2.2 Materials and measures

#### 2.2.1 Mood and IQ measures

Anhedonia and depression symptoms could both potentially influence positive emotional response. Therefore, we assessed whether participants allocated to each condition had comparable levels of depression (using the Beck Depression Inventory-Revised [BDI-II]; Beck, Steer, & Brown, 1996) and anhedonia (using the Snaith Hamilton Pleasure Scale [SHAPS]; Snaith et al., 1995) at intake. Similarly, given that levels of cognitive functioning may impact on how fluently individuals could recall each memory, the National Adult Reading Test (NART; Nelson and Willison, 1991) was used to estimate IQ.

#### 2.2.2 Identification of memories prior to the experimental session

It was decided to select memories to recall prior to attending the testing session to ensure that participants could identify suitable candidate experiences and to minimize any pre-rehearsal of the memories immediately before the experimental manipulations. In e-mail correspondence with the experimenter at least 48 h prior to attending the testing session, participants identified two positive autobiographical memories to recall and a cue word to prompt this remembering. Participants were instructed: “Before participating in the experiment we would like to ask you to remember and briefly describe two personal mildly positive memories (i.e. an experience where you felt positive emotions like happiness, joy, gratitude and/or contentment) and to generate key words that best remind you of them. Please select memories that you would rate as mildly positive. For example, on a scale from zero (not at all positive) to ten (the most positive you have ever felt), select memories in the range of four to seven. We will ask you to think about these memories when you come in for your testing session.” For each memory, participants were asked to “Briefly provide details about the memory (two or three sentences that outline what it involved)” and to “rate how positive this memory was on a scale from zero (not at all) to ten (the most positive you have ever felt)”. Participants were then asked to “Please generate a cue word to remind you of this positive memory”, which was then used in the experimental testing session. While it is possible that the emotion experienced during an activity may differ from the emotion experienced when recalling it, this nevertheless makes it more likely that the two memories participants recall will serve as equivalently effective positive mood inductions. We chose to ask participants for mildly positive memories to avoid ceiling and floor effects in the experiment (where it would not be possible for the experimental manipulations to increase or decrease affective experience during the recall).

#### 2.2.3 Memory recall procedure during experimental session

**Fig. 1:**
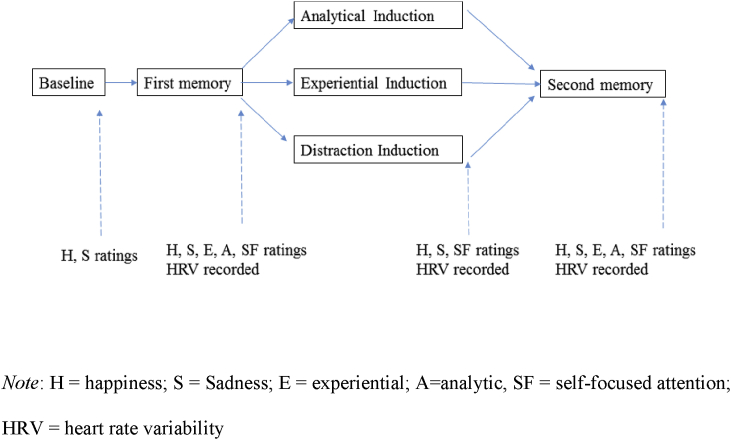
Overview of experimental task.

During the testing session, a bespoke task (programmed in Microsoft Visual Basic 2010) was developed to guide recall of positive autobiographical memories before and after inducing processing mode (see Fig. 1 for illustration of the task). Participants recalled their two positive memories (in random order) in response to the cue word for 5 min each, writing down anything that went through their mind during for the duration of the recall period (written stream of consciousness approach; see Dalgleish, Yiend, Schweizer, & Dunn, 2009). These written narratives made it possible to check whether the subject material participants chose to recall was positive in nature.

#### 2.2.4 Processing mode manipulation

In between the two memory recalls, participants were randomly allocated to an experiential processing mode, analytical processing mode, or a distraction control condition (n = 33 per condition). Each manipulation asked participants to read general statements (unrelated to the content of either memory) to induce a particular processing mode. This processing mode was then intended to carry over into the recall of the second memory. In the experiential and analytical condition participants were asked to read up to 28 self-referential statements about themselves, their current feelings, and physical state (e.g., “how relaxed or agitated you feel” or “the degree of clarity in your thinking right now”) at their own pace, for a total of 8 min (following Watkins & Teasdale, 2004). In the experiential condition, the statements were prefixed with the instruction: “As you read the items, use your imagination and concentration to focus your mind on each experience. Spend a few moments visualizing and concentrating on your experience, attempting to find a phrase, image or set of words that best describes the quality of what you sense.” Each specific item was preceded by the prompt: “Focus your attention on your experience of:”. In the analytical condition participants were instead instructed: “As you read the items, use your imagination and concentration to think about the causes, meanings, and consequences of the statements. Spend a few moments visualizing and concentrating on each statement, attempting to make sense of and understand the issues raised by each statement”. Each specific item was preceded by the prompt: “Think about:”. As the analytical condition is identical to the experiential condition in everything except the instructions about which processing mode to engage in (including the degree to which self-focused attention is induced), it represents a very robust active control. Participants in the distraction control condition read up to 45 items that described a situation with an external focus (e.g., “the structure of a long bridge” or “a truckload of watermelons”) for a total of 8 min at their own pace (adapted from Lyubomirsky & Nolen-Hoeksema, 1993). They were instructed: “As you read the items, use your imagination and concentration to focus your mind on each of the ideas. Spend a few moments visualizing and concentrating on each item”. The distraction condition is intended as a neutral control.

#### 2.2.5 Affect ratings

Participants rated their happiness and sadness experience on single item sliding visual analogue scales ranging from 1 (not at all) to 100 (extremely) at various points of the experimental task. To familiarize themselves with the rating scales, participants first rated their baseline levels of happiness and sadness at the start of the task. Immediately following each memory recall, participants also rated how much they had experienced happiness on sadness on average during the memory recall period.

#### 2.2.6 Manipulation checks

As a manipulation check participants were asked to rate the extent to which they engaged in experiential and analytical processing during each memory recall, on single item scales ranging from 1 (not at all) to 9 (extremely). To rate experiential processing participants were asked to what extent they focused on the sensory experience of the memory during the memory recall. To rate analytical processing, participants were asked to what extent they thought about the causes, meanings and consequences of the memory during the memory recall.

To rule out the possibility that it was a change in the external versus internal focus of attention driving any observed condition differences, we additionally asked participants to rate how self or externally focused they felt on a single item scale of 1 (entirely self-focused) to 9 (entirely externally focused) during each memory recall.

To check whether the experiential, analytical and distraction inductions impacted on mood and self-focus in their own right, participants rated on average how happy and sad (using the same single item 100 point rating scales described above) and self-focused (using the same 9 point single item rating scale described above) they had felt during the inductions.

The written narratives generated during each memory were inspected by an independent rater (blind to experimental condition) to check whether participants had chosen to recall a positive experience. For five participants it was not obvious that the memories generated were positive in nature. Therefore, we repeated all analyses excluding these individuals.

#### 2.2.7 Psychophysiological recording

Heart rate was recorded continuously throughout the experimental task using a BIOPAC MP100 system (BIOPAC, 1997) acquiring data at 1000 samples per second. The raw electrocardiogram (ECG) signal was measured by attaching two disposable Ag-AgCL electrodes on the dorsal forearms with clip-on shielded leads and an additional ground lead. Log respiratory sinus arrhythmia was used to index HRV, which has been argued to be a sensitive index of (sympathetic) vagal tone relating to positive affect (Allen et al., 2007, Porges, 2007). Log respiratory sinus arrhythmia (henceforth referred to as HRV) during each recall period and during the processing mode manipulation was computed using CMetX software (Allen et al., 2007), having first inspected and cleaned the data to remove any recording artefacts that could bias HRV estimation. Four participants’ data were excluded from the HRV analyses due to equipment failure meaning that no ECG trace was recorded.

### 2.3 Procedure

Participants were sent an information sheet via e-mail. If they provided e-mail consent to take part, they were then sent instructions to identify two positive autobiographical memories to recall prior to attending the testing session. The 1 h experimental session was conducted in a private, quiet testing cubicle at the MRC CBU. Consent was confirmed face-to-face; demographic information and baseline measures were collected; the ECG electrodes were attached; and then participants completed the experimental task. Participants received an honorarium of £6 per hour for their time and £3 to refund travel expenses.

## 3 Results

All analyses were two-tailed with alpha set at 0.05. Inspection of the data found that happiness, experiential processing, analytical processing and HRV were satisfactorily normally distributed. However, sadness was positively skewed and could not be corrected using transformation, so the sadness data were rank transformed in all subsequent analyses (non-parametric bridging; Conover & Iman, 1982).

### 3.1 Mood manipulation check analyses

To examine whether the positive memory recall was an effective mood manipulation, happiness change during the first memory recall was assessed (i.e. prior to any processing mode manipulation taking place). A repeated measure analysis of variance (ANOVA) (collapsing across the three experimental conditions) revealed a significant increase in happiness from the baseline phase (M = 60.21, SD = 15.65) to the memory recall phase (M = 71.70, SD = 16.97), *F*(1,98) = 55.36, *p* < 0.01, η _p_^2^ = 0.36. Comparable analysis on sadness ratings found no significant decrease from baseline (M = 7.30, SD = 11.44) to recall (M = 8.81, SD = 12.75), *F*(1,98) = 1.28, *p* = 0.26, η _p_^2^ = 0.01. Therefore, the manipulation successfully increased happiness but had no impact on sadness as intended.

### 3.2 Relationship between spontaneous processing mode and response to the first memory

Hypothesis One predicted that greater spontaneous use of an experiential processing mode during the first memory recall would be associated with greater happiness experience and greater HRV. To test this prediction, a series of Pearson's correlations were run on responses during the first memory. Partially consistent with Hypothesis One, greater happiness experience was significantly associated with greater experiential ratings (M = 5.23, SD = 2.29), *r* = 0.26, *p* < 0.01. Happiness experience was not significantly associated with analytical (M = 4.46, SD = 2.66), *r* = -0.03, *p* = 0.77, or self-focus (M = 2.64, SD = 2.24), *r* = 0.16, *p* = 0.12, ratings. HRV was not significantly related to experiential, *r* = -0.17, *p* = 0.10, analytical, *r* = -0.05, *p* = 0.66, or self-focus, *r* = -0.06, *p* = 0.59, ratings, failing to support Hypothesis One. Exploratory analyses found that sadness experience was not significantly related to experiential, *r* = -0.02, *p* = 0.88, analytical, *r* = 0.03, *p* = 0.76, or self-focus, *r* = -0.04, *p* = 0.72, ratings.

### 3.3 Baseline and manipulation check analyses

Before testing Hypothesis Two, the data were examined to determine whether the groups were comparable at baseline and whether the processing mode induction had been successful. Table 1 reports baseline clinical and demographic characteristics of participants in each condition. Baseline measures were analyzed with a series of univariate ANOVAs for continuous variables and chi-squared tests for categorical variables. As intended, there were no significant differences between conditions on the baseline measures (including depression and anhedonia severity), *Fs <* 1.36, *p*s > 0.26.

**Fig. 2:**
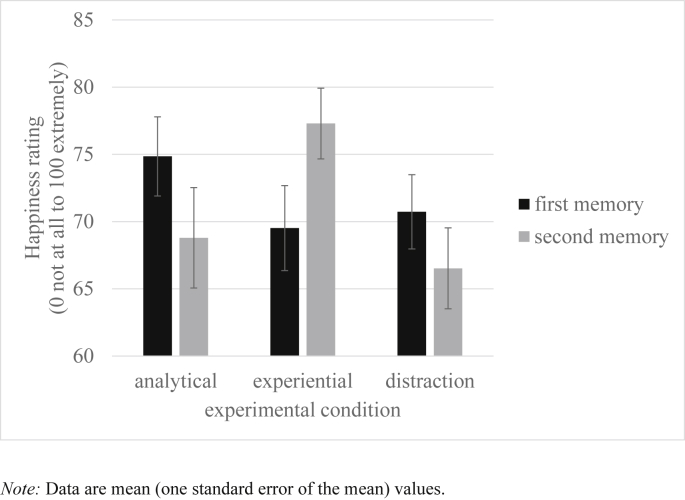
Happiness experience during each memory recall in the analytical, experiential and distraction conditions.

**Fig. 3:**
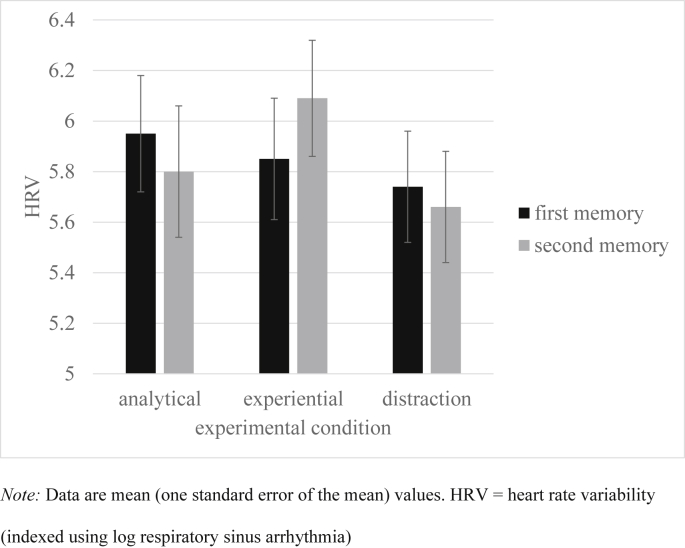
HRV during each memory recall in the analytical, experiential and distraction conditions.

**Fig. 4:**
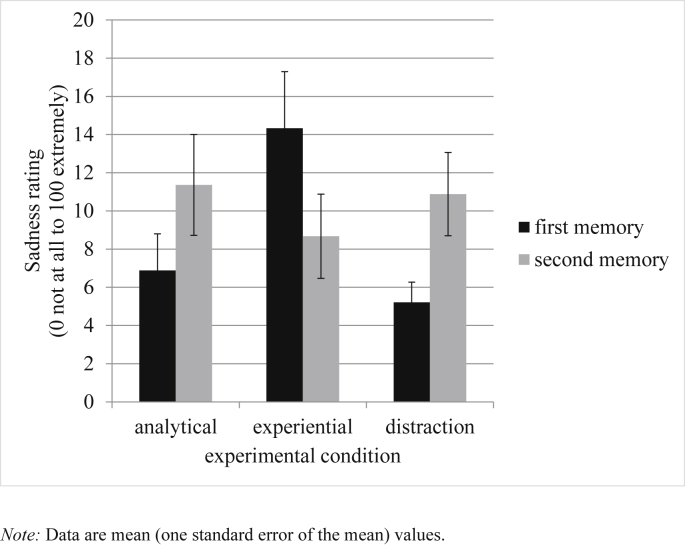
Sadness experience during each memory recall in the analytical, experiential and distraction conditions.

Similarly, univariate ANOVAs examined if response to the first memory differed between conditions. Table 2 reports experiential, analytical and self-focus ratings during the pre- and post- processing mode induction memory recall. Fig. 2, Fig. 3, Fig. 4 plot happiness ratings, HRV and sadness ratings during each memory recall respectively. As intended, participants across the three conditions did not differ in self-reported happiness, HRV, experiential ratings, or self-focus ratings during the first memory, *F*s < 1. However, sadness ratings to the first memory differed between conditions at the level of a non-significant trend, *F*(2,96) = 2.55, *p* = 0.08, η _p_^2^ = 0.05. Pairwise comparisons revealed that the experiential condition experienced more sadness than the analytical, *p* = 0.06, and distraction, *p* = 0.05, condition (both at the level of a non-significant trend). The analytical and distraction conditions did not differ in sadness ratings, *p* = 0.97. Moreover, analytical ratings during the first memory significantly differed between conditions, *F*(2,96) = 3.53, *p* = 0.03, η _p_^2^ = 0.07. Pairwise comparisons found that individuals in the distraction condition were more analytical than those in the experiential condition, *p* < 0.01, but no other differences were significant, *p*s > 0.17.

To assess the success of the processing mode manipulation, changes in experiential processing, analytical processing and self-focused attention ratings during memory recall were analyzed (see Table 2). An analysis of covariance (ANCOVA) approach was used to take into account any condition differences in ratings during the first memory. In each case, the rating that was taken during the second memory recall was the dependent variable; condition (experiential, analytical, control) was the between-subjects factor; and the rating taken during the first memory recall was the covariate. As intended, there was a significant effect of condition on experiential ratings, *F*(2,95) = 4.02, *p* = 0.02, ${{\text{η}}_{p}}^{2}$ = 0.08. Pairwise comparisons showed that experiential ratings were greater in the experiential condition relative to the analytical, *p* < 0.01, and distraction, *p* = 0.04, conditions, while the analytical and distraction conditions did not differ from one another, *p* = 0.58. There were no differences between conditions for analytical or self-focus ratings, *F*s < 1.

To examine the possibility that the processing mode induction served as a mood and self-focus manipulation in its own right, ratings taken immediately after the induction were also analyzed using univariate ANOVAs (see Table 2). As intended, there were no condition differences in happiness experience or HRV during the processing mode induction, *Fs* < 1. However, the conditions differed for sadness ratings during the induction, *F*(2,96) = 3.73, *p* = 0.03, ${{\text{η}}_{p}}^{2}$ = 0.07. The distraction condition induced less sadness than either the analytical or experiential condition, *ps* < 0.05, which in turn did not differ from one another, *p* = 0.56. Moreover, there was a significant difference between conditions for self-focus ratings during the induction, *F*(2,96) = 32.45, *p* < 0.001, with pairwise comparisons showing that the distraction condition reported less self-focused attention than both the analytical and experiential conditions, *ps* < 0.001. The analytical and experiential conditions did not differ in levels of self-focused attention, *p* = 0.81. This pattern of findings indicates that any condition differences in happiness experience during the second memory recall are not simply an artifact of altered happiness experience during the processing mode inductions carrying over into the recall task. Differences in levels of self-focused attention and sadness could potentially account for differences between the distraction condition and the other two conditions, but critically not between the experiential and analytical conditions.

### 3.4 Impact of processing mode manipulation on emotional response

Hypothesis Two predicted that the induction of an experiential processing mode would result in increased happiness experience and greater heart rate variability during the positive memory recall, relative to both the analytical and distraction comparison conditions. To test this prediction an ANCOVA approach was again followed, with ratings during the second memory recall as the dependent variables; condition as the between-subjects factor, and ratings taken during the first memory recall as the covariate. Supporting predictions, analyses found that happiness ratings, *F*(2,95) = 7.43, *p* < 0.01, η _p_^2^ = 0.14, and HRV, *F*(2,95) = 3.90, *p* = 0.02, η _p_^2^ = 0.08, during the positive mood manipulation varied significantly across conditions (see Fig. 2, Fig. 3). Pairwise comparisons revealed that the experiential group differed from the analytical, *p* < 0.01, and distraction, *p* < 0.01, conditions in experienced happiness. Moreover, HRV differed in the experiential group relative to the both the analytical, *p* = 0.01, and distraction, *p* = 0.03, control conditions. The distraction and analytical conditions did not significantly differ for happiness ratings or HRV, *p*s > 0.71. To further interrogate these findings, paired sample t-tests examined whether there was a significant change in happiness experience and HRV from the first to the second memory for each condition separately. For the experiential condition, there was a significant increase in happiness experience, *t*(32) = 2.58, *p* = 0.02, Cohen's d (Cohen, 1992) = 0.47, and a trend significant increase in HRV, *t*(31) = 1.89, *p* = 0.07, d = 0.18. For the analytical condition, there was a significant decrease in happiness experience, *t*(32) = 2.28, *p* = 0.03, d = 0.31, and a non-significant decrease in HRV, *t*(30) = 1.65, *p* = 0.11, d = 0.10. The distraction control condition showed a decrease in happiness experience at the level of a non-significant trend, *t*(32) = 1.83, *p* = 0.08, d = 0.26, and a non-significant decrease in HRV, *t* < 1. These findings broadly support the second hypothesis in that happiness experience and HRV increased from the first to second memory in the experiential condition (relative to both the analytical and distraction conditions). We also ran exploratory ANCOVA analyses of sadness ratings and found no difference between conditions, *F*(2,95) = 1.50, *p* = 0.23, ${{\text{η}}_{p}}^{2}$ = 0.03 (see Fig. 4).

### 3.5 Further exploratory analyses

To rule out whether baseline differences in spontaneous use of analytical processing were contaminating results, we repeated the happiness experience, HRV and sadness experience manipulation analyses when additionally entering analytic ratings during the first memory recall as a covariate. We also repeated these analyses when excluding the five participants whose memories were not clearly positive in nature (one participant in the analytical condition where both memories were not positive, one participant in both the distraction analytical conditions where the second memory was not positive, and two participants in the experiential condition where the second memory was not positive). Finally, we reran the happiness and sadness analyses using a residual change score approach (regressing ratings of the baseline onto the first memory ratings and ratings during the processing mode induction onto the second memory ratings). In all cases, an identical pattern of findings emerged (see Supplementary Material Table S1). The experiential manipulation lead to an increase in happiness experience and HRV from the first to the second memory, relative to the analytical and control conditions. There was no impact on sadness ratings. Therefore, baseline differences in spontaneous use of analytical processing, including people who did not recall clearly positive memories, and using absolute rather than change score analyses are not influencing the observed pattern of results. Finally, we repeated all analyses when including depression severity as a continuous covariate, to see if depression moderated the pattern of findings (see Supplementary Material Section Two). These analyses found that the benefits of experiential processing were not diminished (and may even be enhanced) with increasing symptoms of depression, although these findings need to be interpreted cautiously given the non-clinical nature of the sample used here.

## 4 Discussion

The present study examined whether inducing an experiential processing mode enhances positive emotional response during positive autobiographical memory recall. Participants recalled two positive autobiographical memories, the first before and the second after being randomized to an experiential processing mode, analytical processing mode, or distraction manipulation.

Partially supporting Hypothesis One, greater spontaneous use of an experiential processing mode during the first memory recall (prior to processing mode being induced) was associated with greater subjective happiness ratings. However, experiential processing mode induction was unrelated to HRV during the first memory recall. As intended, the experiential processing mode induction increased levels of experiential processing from the first to the second memory, to a greater extent than the analytical and control conditions. However, the analytical processing mode induction did not increase levels of analytical processing from the first to the second memory any more than the other experimental conditions. Supporting Hypothesis Two, HRV response differed between conditions. HRV increased from the first to second memory in the experiential condition (marginally significant; a small effect size; Cohen, 1992) and decreased from the first to the second memory (not significant; a small effect size) in the other two conditions. No significant differences were found between the analytical and distraction conditions in any analyses.

The extant literature has generally found that experiential processing bolsters positive affect during positive activities (Holmes et al., 2006, Holmes et al., 2009), but its impact on positive affect during positive memory recall is less clear cut (Hetherington and Moulds, 2015, Nelis et al., 2015). The present study refined a number of methodological features of these previous memory studies, including applying a mixed within- and between-subjects design to increase sensitivity, including a neutral control condition to help determine the direction of effects, and recording objective psychophysiological indices of positive affect. After making these refinements, a clear link was observed between experiential processing and positive emotional response. In particular, it was possible to demonstrate that inducing experiential processing led to an *increase* in positive affect from the first to the second memory (rather than that analytical processing decreased positive affect). This effect was clearly demonstrated in terms of subjective happiness report. HRV analyses also indicated a similar pattern, although the effect size was smaller and the significant interaction between conditions was carried by a trend significant *increase* in the experiential condition relative to a non-significant *decrease* in the other conditions. That comparable findings emerged using objective HRV measures is encouraging, as these are less susceptible to demand effects than self-report ratings of happiness. Suggesting that these findings are robust, there was a triangulation between the findings that spontaneous use of experiential processing was linked to happiness experience to the first memory and that inducing experiential processing led to an increase in happiness experience from the first to the second memory.

It is important to comment on the fact that the analytical manipulation did not reliably increase analytical processing in the present study, in contrast to many previous studies in the literature (for example, Watkins et al., 2008, Watkins and Teasdale, 2001, Watkins and Teasdale, 2004). It may be that the measure of analytical processing used in the present study was not sufficiently sensitive to detect change. Alternatively, it may indicate that this manipulation is less robust than previously assumed. For example, it is possible that the instruction to ‘use your imagination and concentration to think about the causes, meanings and consequences of the statements’ induces a visual method of recall that counteracts the effects of abstract processing. However the analytical condition is interpreted, it nevertheless constitutes a carefully matched control for the experiential condition given that it was identical in every way apart from the processing mode instructions.

Exploratory analyses found no association between sadness ratings and spontaneous use of experiential and analytical processing during the first memory and no impact of experimentally inducing experiential and analytical processing on sadness ratings during the second memory. This suggests that processing mode does not influence the extent to which individuals experience negative affect using positive memory recall. This may in part reflect the fact that individuals were close to floor in levels of negative affect prior to the memory recall, so it was not possible to lower this further.

Overall, the present findings support the general thesis that encouraging experiential processing can bolster positive emotional response. These results tentatively suggest that existing psychological intervention elements targeting experiential processing in mindfulness, behavioral activation, imagery, and positive psychology savoring interventions (Blackwell et al., 2015, Bryant and Veroff, 2007, Martell et al., 2010, Williams, 2008) are likely to be of benefit to clients by enhancing positive mood. These findings now require replication and extension in clinical populations before they are used to guide intervention strategy.

There are also a number of other ways to extend these findings. First, while psychophysiology measures are less likely to be strongly biased by demand effects than self-report data, they are not sufficient to completely rule out the possibility that demand effects were contaminating the present results. Future studies could explicitly ask participants about the beliefs they held about the likely impact of each processing mode on positive emotion experience in order to assess directly demand characteristics. Second, it would be useful to unpack further which specific aspects of experiential processing enhance positive emotional response. For example, experiential processing consists of a number of different elements of dispositional mindfulness, including keeping the mind in the moment (present moment awareness), not evaluating experience as it unfolds (non-judgement), and attending to sensory experience (observing) (for an overview of different facets of dispositional mindfulness, see Baer, Smith, Hopkins, Krietemeyer, & Toney, 2006). Finally, it is important to examine whether the nature of the positive memory recalled modifies the impact of experiential processing on affective experience. For example, it has recently been demonstrated that depressed individuals are unable to repair negative moods using positive memory recall if the memory is not concordant with their current sense of self (Werner-Seidler, Tan, & Dalgleish, 2016). It is conceivable that the impact of experiential processing on affective experience could be moderated by how self-concordant the memory being recalled is.

A number of limitations need to be considered about the present study. First, we cannot rule out that participants actively rehearsed the memories in the days after they had generated them and before the testing session. In the event that the participants did rehearse their memories, rehearsal effects are likely to have been short lived and not extended into the testing session. Importantly, any such effects would not have differentially impacted on the analytical versus experiential processing mode manipulations. Second, we assessed happiness and sadness with single item visual analogue scales, consistent with our previous studies in this area (Dunn et al., 2004, Dunn et al., 2009). It is possible that use of multiple item rating scales (for example, use of the Positive and Negative Affect Scale to index positive and negative mood; Watson, Clark, & Tellegen, 1988) may have been more sensitive. However, we chose to use a single item scale to minimize participant burden and to avoid having lengthy delays rating multiple items between each phase of the experiment. Third, we excluded participants on the basis of them having a current diagnosis of mental health condition. This assessment would have been more robust if following a structured clinical interview to guide diagnosis (e.g. First, Spitzer, Gibbon, & Williams, 1996). Fourth, the within-subjects design (where participants each recall two memories, the second of which involved manipulation of processing mode) is both a strength and a weakness of the present study. On the one hand, it controls for individual differences in affective response to memories, potentially increasing sensitivity of the analyses. On the other hand, it is possible that participants may habituate to the task during the second recall. However, we would not expect this habituation to differentially influence the experimental conditions, so this cannot account for the observed condition differences. Fifth, as discussed previously, the analytical condition failed to induce analytical processing. This does not undermine the key findings in the present study about the impact of experiential processing on affective experience (relative to two control conditions). However, it does mean that no conclusions can be drawn about the impact of analytical processing on affective experience during positive memory recall. Further research using a more robust analytical induction is required to answer this question.

In summary, the present study demonstrates that engaging in an experiential processing mode can bolster positive emotional response. It is now important to replicate this finding in a clinical sample to determine whether including experiential exercises in existing psychological interventions could help repair anhedonia in major depressive disorder and other clinical groups characterized by reduced pleasure experience.

## Figures and Tables

**Table 1:** Clinical and demographic characteristics of the sample and response during the processing mood induction broken down by condition.

Variable	Condition		
	Analytic	Experiential	Distraction
	Mean (SD)	Mean (SD)	Mean (SD)
Gender (M/F)	13/20	14/19	13/20
Age	32.83 (12.94)	31.94 (11.61)	37.21 (16.43)
Estimated Full Scale IQ	116.64 (7.53)	117.54 (4.30)	117.64 (5.03)
Ethnicity (% Caucasian)	84.8%	87.9%	84.8%
Mental Health History (Y/N)	1/32	1/32	2/31
BDI-II	5.03 (4.70)	5.30 (4.35)	4.85 (4.65)
SHAPS	18.82 (3.97)	18.55 (6.44)	19.52 (4.72)
Happiness at baseline	61.73 (18.03)	62.09 (12.29)	56.82 (15.99)
Sadness at baseline	7.21 (12.54)	8.06 (11.99)	6.64 (9.92)

*Note.* BDI-II=Beck Depression Inventory-II; SHAPS=Snaith-Hamilton Pleasure Scale.

**Table 2:** Manipulation check variables during memory recall before and after the processing mode induction for participants in each condition.

Variables		Condition		
		Analytical	Experiential	Distraction
First memory	Experiential ratings	5.18 (2.20)	5.52 (2.02)	5.00 (2.63)
	Analytical ratings	4.48 (2.85)	5.30 (2.31)	3.61 (2.60)
	Self-focus ratings	2.91 (2.48)	2.64 (2.07)	2.36 (2.19)
Induction	Happiness ratings	52.61 (24.52)	57.36 (20.88)	58.03 (19.99)
	Sadness ratings	14.94 (17.04)	17.27 (17.53)	6.39 (7.10)
	Self-focus ratings	1.55 (1.25)	1.67 (1.38)	5.18 (3.09)
	HRV	6.24 (1.12)	6.19 (1.07)	6.19 (1.12)
Second memory	Experiential ratings	4.91 (1.99)	6.24 (1.79)	5.12 (2.12)
	Analytical ratings	4.82 (2.11)	4.85 (2.33)	4.27 (2.35)
	Self-focus ratings	3.33 (2.03)	3.12 (2.40)	3.15 (2.67)

*Note:* Data are mean (standard deviation) values. First memory = memory recall before the processing mode induction; second memory = memory recall after the processing mode induction. Induction = experiential, analytical or distraction processing mode induction in between each memory recall. HRV = heart rate variability (indexed using log respiratory sinus arrhythmia).

## Footnotes

1 Study Two in Nelis et al. (2015) included a third condition. This was intended to lower positive emotional response by encouraging participants to make an unfavourable verbal comparison between their current state and this past happy state. As expected, participants in the verbal comparison condition showed less of an increase in positive mood when recalling the memory, relative to the both the analytical and concrete conditions (who did not differ from one another).
