# Supplementary material for: Engaging in an experiential processing mode increases positive emotional response during recall of pleasant autobiographical memories
Source: Behav Res Ther. 2017 May;92:68–76. doi: 10.1016/j.brat.2017.02.005 (PMC5390771; doi:10.1016/j.brat.2017.02.005)
Supplement: Online data [file mmc1.docx]

**Supplementary materials to Gadeikis et al submission**

Table S1 reports results of additional analyses, all of which support the conclusion that experiential processing mode bolsters happiness reactivity and HRV response during positive autobiographical memory recall, relative to the analytical and distraction control conditions.

Table S1.

Additional analyses: i) excluding five people who did not clearly recall positive memories, ii) controlling for analytical ratings to first memory, and iii) using residual change scores

| Description | Variable | Main effect of condition | E > A | E > D | A > D |
| --- | --- | --- | --- | --- | --- |
| Excluding five people who did not clearly recall positive memories | Happiness | *F*(2,90)=5.81, *p*<.01 | *F*(1,61)=7.81, *p*<.01 | *F*(1,60)=9.42, *p*<.01 | *F*<1 |
|  | HRV | *F*(2,86)=3.50, *p*=.03 | *F*(1,56)=5.26, *p*=.03 | *F*(1,58) = 4.11, *p*<.05 | *F*<1 |
|  | Sadness | *F*<1 | *-* | *-* | *-* |
| Controlling for analytical ratings to first memory | Happiness | *F*(2,94)=6.18, *p*<.01 | *F*(1,62)=8.64, *p*<.01 | *F*(1,62)=8.54, *p*<.01 | *F*<1 |
|  | HRV | *F*(2,90)=3.88, *p*=.02 | *F*(1,59)=5.70, *p*=.02 | *F*(1,60)=3.90, *p*=.05 | *F*<1 |
|  | Sadness | *F*(2,94)=1.21, *p*=.30 | *-* | *-* | *-* |
| Using residual change scores | Happiness | *F*(2,95)=5.79, *p*<.01 | *F*(1,63)=4.58, *p*=.04 | *F*(1,63)=11.44, *p*<.01 | *F*<1 |
|  | Sadness | *F*(2,95)=1.95, *p*=.15 | *-* | *-* | *-* |

*Note:* Pairwise comparisons only reported where main effect of condition was significant. E>A=result of pairwise comparison of experiential and analytical condition; E>D= results of pairwise comparison of experiential and distraction condition; A>D= result of pairwise comparison of analytical and distraction condition. Residual change score analyses not conducted for HRV, as this was only computed during each memory recall.

Depression moderation analyses

There is evidence from other domains that the same processing style can have different effects as a function of symptom severity. For example, following prompts to ruminate tends to have a more detrimental effect in individuals who are currently depressed or dysphoric compared to those who are not (e.g. see Watkins, 2008; Lyubomirsky et al., 2015). Therefore, it is important to examine if depression severity also moderates responses to experiential processing mode inductions.

We conducted exploratory analyses to examine if depression severity (BDI-II scores) moderated the pattern of findings observed in both the spontaneous and the manipulation analyses. These analyses need to be interpreted cautiously given that there was relatively little range in depression severity across the sample (mean BDI-II=5.07, SD=4.53, range=0-18).

For the spontaneous analyses, depression severity and spontaneous use of experiential processing during the first memory were mean centred and an interaction term was computed from these mean centred variables. A linear regression was then run to predict happiness experience, HRV and sadness experience, entering depression severity and spontaneous experiential processing (both mean centred) at step one of the model and the interaction term at step two. Moderation is indicated by model fit improving from step one to step two.

For happiness experience, step one of the model was significant, Δ*F* (2,96)=5.93, *p*<.01, Δr^2^ =.11. Inspection of the coefficients revealed that greater happiness experience to the first memory was independently associated with less depression severity, *r_p_*=-.21, *p*=.04, and greater experiential focus, *r_p_*=.25, p=.01. Adding the interaction term at step two did not improve model fit, Δ*F* <1, indicating that depression severity did not moderate the association between spontaneous use of experiential processing and happiness experience. For HRV analyses, neither step one, Δ*F*(2,92) =1.48, *p*=.23, Δr^2^ =.03, nor step two, Δ*F*<1, of the model were significant. For sadness experience, again neither step one, Δ*F*(2,96) =1.41, *p*=.25, Δr^2^ =.03, nor step two, Δ*F*(2,92) =2.76, *p*=.10, Δr^2^ =.03, of the model reached significance. This indicates depression severity is unrelated to the pattern of HRV and sadness experience data observed during the first memory.

To analyse depression moderation in the manipulation analyses, the residual change in happiness experience, HRV and (rank transformed) sadness experience were computed by regressing pre-ratings onto post-ratings and saving the unstandardized residual as a new variable. These residual change scores were then analyzed in ANCOVAs, with condition as the between-subjects factor and (mean centred) depression severity as a continuous covariate. If depression is moderating the pattern of findings, the interaction term between depression severity and condition should be significant. For happiness experience, there was no significant main effect of depression severity, *F*<1, but there was a significant interaction between depression severity and condition*, F*(2,93)=3.75, *p*=.03, η _p_² =.08. For HRV, there was no significant main effect of depression severity, *F*<1, and no significant interaction between depression severity and condition, *F*(2,89)=1.33, *p*=.27, η _p_² =.03. For sadness experience, the main effect of depression severity did not reach significance, *F*(2,93)=1.54, *p*=.22, η _p_² =.02, and nor did the interaction between depression severity and condition, *F*(2,89)=2.21, *p*=.12, η _p_² =.05. To resolve the happiness experience interaction we reran the analyses on each pair of conditions separately. The interaction term was significant when contrasting the experiential and analytical conditions, *F*(1,62)=5.16, *p*=.03, η _p_² =.08, and the experiential and distraction conditions, *F*(1,62) = 6.63, *p*=.01, η _p_² =.10, but not the analytical and distraction conditions, *F*<1.

Further, we correlated depression severity with the residual change in happiness experience from the first to the second memory in each condition separately. In the experiential condition, greater depression severity was associated with non-significantly *greater* happiness experience change, *r*=.29, *p*=.10, whereas in the analytical and distraction conditions, greater depression severity was associated with non-significantly *smaller* happiness experience change (*r*=-.27, *p*=.13 and *r*=-.33, *p*=.06 respectively). In other words, increasing levels of depression severity were associated with participants showing a pattern of benefiting more from the experiential manipulation and benefiting less from the analytical and distraction manipulations.

A number of tentative comments can be made on the basis of these analyses. First, higher levels of depressive symptomatology were associated with less happiness experience during the first memory recall, consistent with previous findings that depression is associated with reduced response to positive laboratory manipulations (for example, Dunn, Dalgleish, Lawrence, Cusack, & Ogilvie, 2004; Rottenberg, Kasch, Gross, & Gotlib, 2002; see meta-analysis by Bylsma, Morris, & Rottenberg, 2008). Depression severity was unrelated to HRV during the first memory, which is also consistent with the finding that reductions in positive emotional response in depression are more robustly demonstrated using self-report than psychophysiological measures (Dunn, 2012).

Second, depression severity to some extent moderated the effects of the processing mode induction on happiness experience (but not HRV). In particular, greater levels of depression were associated with a greater happiness increase during the experiential processing mode induction. Conversely, increasing levels of depression were associated with a reduced happiness increase during the analytical and distraction control condition. While none of the associations between depression and happiness reactivity were individually significant within a condition, the pattern of these associations did significantly vary between conditions. The experiential findings are tentatively consistent with the view that more depressed individuals will gain more benefit from experiential processing. Given that the analytical induction did not reliably increase analytical processing and that the distraction condition was intended to be a neutral control, one way to interpret these analytical and control findings is that more depressed individuals simply habituated more rapidly to the positive memory recall task.

It is interesting to contrast these moderation findings to the previous studies in this area. Hetherington & Moulds (2013) intentionally recruited high and low dysphoric groups and examined how dysphoria status moderated results, finding that group status did not moderate the impact of processing mode inductions on happiness experience. Nelis et al. (2015) found that depression severity (modelled continuously in a community sample) did not moderate any differential impact of experiential versus analytical inductions on positive experience.

Overall, the present findings support the general thesis that encouraging experiential processing can bolster positive emotional response. These effects are not diminished (and may even be enhanced) with increasing depression severity. These findings now require replication and extension in a population with more marked variation in depression severity (including those who would meet diagnostic criteria for a current Major Depressive Episode) before they are used to guide intervention strategy in clinical groups. For example, it is conceivable that a curvilinear relationship could exist, whereby experiential processing enhances positive experience in those with mild depression but is harmful in those with severe depression symptoms. Moreover, it would also be interesting to examine if experiential processing mode is a helpful form of positive emotion regulation in other clinical groups characterized by anhedonia (including social phobia and schizophrenia; Kashdan et al., 2011; Watson & Naragon-Gainey, 2010).

**References**

Bylsma, L. M., Morris, B. H., & Rottenberg, J. (2008). A meta-analysis of emotional reactivity in major depressive disorder. *Clinical Psychology Review*, *28*(4), 676–691. <http://doi.org/10.1016/j.cpr.2007.10.001>

Dunn, B. D. (2012). Helping depressed clients reconnect to positive emotion experience: Current insights and future directions. *Clinical Psychology & Psychotherapy*, *19*(4), 326–340. <http://doi.org/10.1002/cpp.1799>

Dunn, B. D., Dalgleish, T., Lawrence, A. D., Cusack, R., & Ogilvie, A. D. (2004). Categorical and dimensional reports of experienced affect to emotion-inducing pictures in depression. *Journal of Abnormal Psychology*, *113*(4), 654–660. <http://doi.org/http://dx.doi.org/10.1037/0021-843X.113.4.654>

Kashdan, T. B., Weeks, J. W., & Savostyanova, A. A. (2011). Whether, how, and when social anxiety shapes positive experiences and events: A self-regulatory framework and treatment implications. *Clinical Psychology Review*, *31*(5), 786–799. <http://doi.org/10.1016/j.cpr.2011.03.012>

Lyubomirsky, S., Layous, K., Chancellor, J., & Nelson, S. K. (2015) Thinking about rumination: The scholarly contributions and intellectual legacy of Susan Nolen-Hoeksema. *Annual Review of Clinical Psychology, 11*, 1-22. http://doi.org/10.1146/annurev-clinpsy-032814-112733

Rottenberg, J., Kasch, K. L., Gross, J. J., & Gotlib, I. H. (2002). Sadness and amusement reactivity differentially predict concurrent and prospective functioning in major depressive disorder. *Emotion*, *2*(2), 135–146. <http://doi.org/10.1037/1528-3542.2.2.135>

Watkins, E. R. (2008) Constructuve and unconstructive repretitive thought. *Psychological Bulletin, 134*, 163-206. http://doi.org/[10.1037/0033-2909.134.2.163](https://dx.doi.org/10.1037%2F0033-2909.134.2.163)

Watson, D., & Naragon-Gainey, K. (2010). On the specificity of positive emotional dysfunction in psychopathology: Evidence from the mood and anxiety disorders and schizophrenia/schizotypy. *Clinical Psychology Review*, *30*(7), 839–848. <http://doi.org/10.1016/j.cpr.2009.11.002>
